# Supplementary material for: Sex differences in oncogenic mutational processes
Source: Nat Commun. 2020 Aug 28;11:4330. doi: 10.1038/s41467-020-17359-2 (PMC7455744; doi:10.1038/s41467-020-17359-2)
Supplement: Supplementary file 1 — Supplementary Information [file 41467_2020_17359_MOESM1_ESM.pdf]

Supplementary Information to

Sex Differences in Oncogenic Mutational Processes

Li *et al.*

Supplementary Figures 1-13

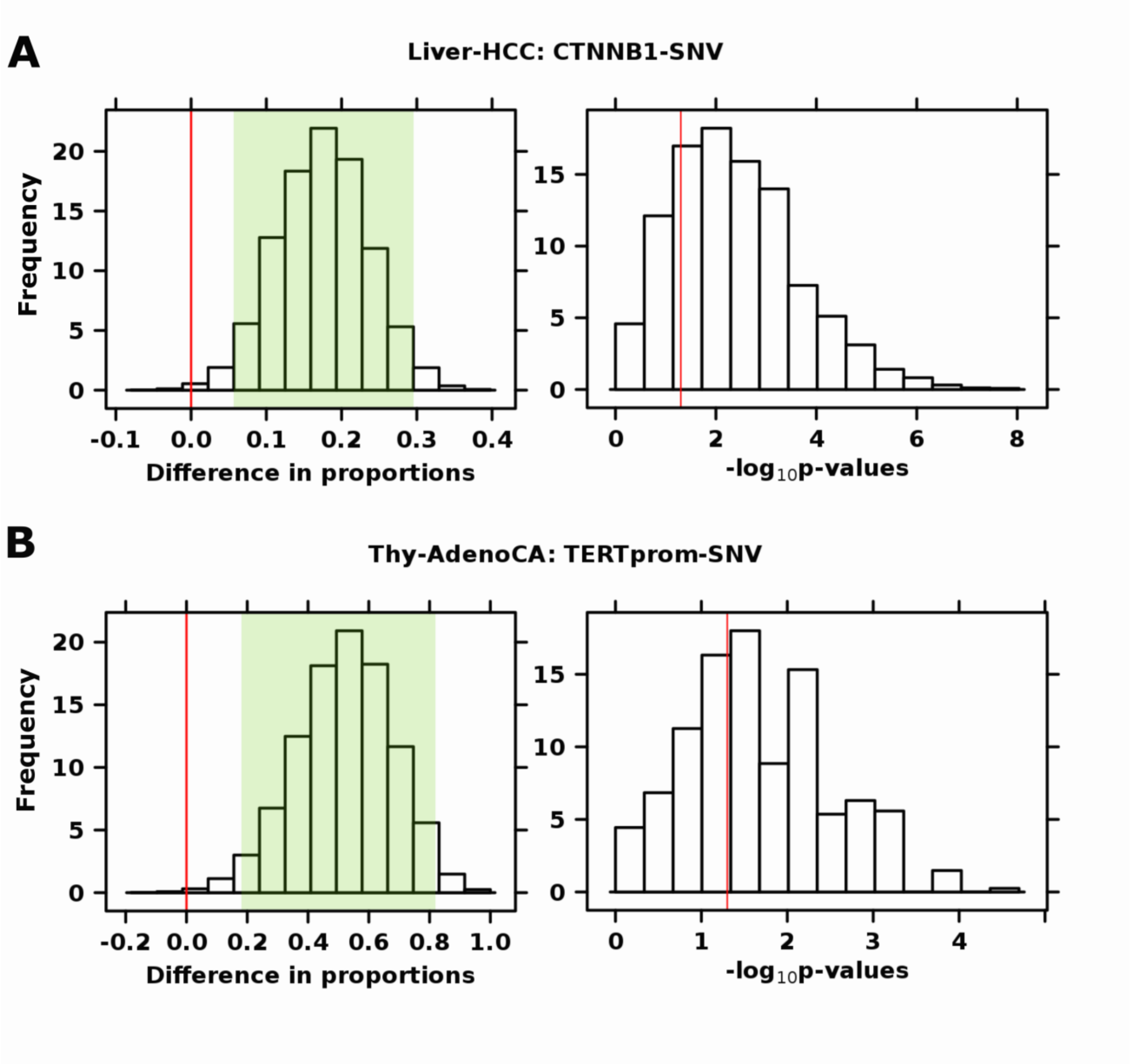

Supplementary Figure 1. Repeated down-sampling on sex for driver SNV analysis.
Results from repeated down-sampling analysis of driver SNV findings to evaluate effect of imbalanced sex. For each set of plots, the left plot shows distribution of differences between male and female proportions with green region indicating 95% confidence interval and red line indicating equal effects between the sexes. Right plot shows p-value distribution from proportions tests with red line showing a p=0.05 threshold. Down-sampling was repeated 10,000 times. **(A)** Down-sampling results for *CTNNB1* mutation in hepatocellular cancer. **(B)** Down-sampling results for *TERT* promoter mutation in thyroid cancer.

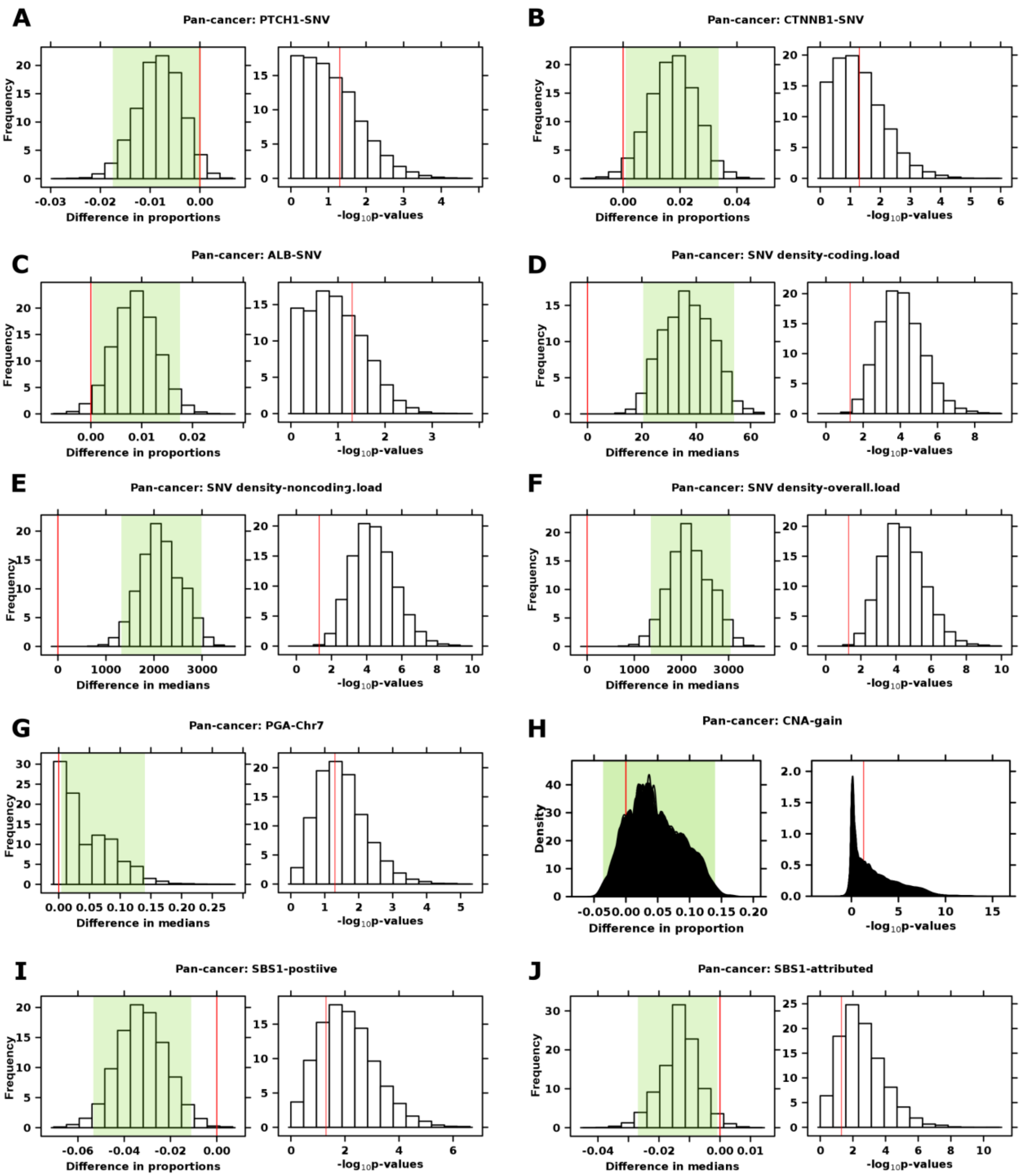
A
Pan-cancer: PTCH1-SNV
Frequency
Difference in proportions
-log10p-values
B
Pan-cancer: CTNNB1-SNV
Frequency
Difference in proportions
-log10p-values
C
Pan-cancer: ALB-SNV
Frequency
Difference in proportions
-log10p-values
D
Pan-cancer: SNV density-coding.load
Frequency
Difference in medians
-log10p-values
E
Pan-cancer: SNV density-noncoding.load
Frequency
Difference in medians
-log10p-values
F
Pan-cancer: SNV density-overall.load
Frequency
Difference in medians
-log10p-values
G
Pan-cancer: PGA-Chr7
Frequency
Difference in medians
-log10p-values
H
Pan-cancer: CNA-gain
Density
Difference in proportion
-log10p-values
I
Pan-cancer: SBS1-postiive
Frequency
Difference in proportions
-log10p-values
J
Pan-cancer: SBS1-attributed
Frequency
Difference in medians
-log10p-values

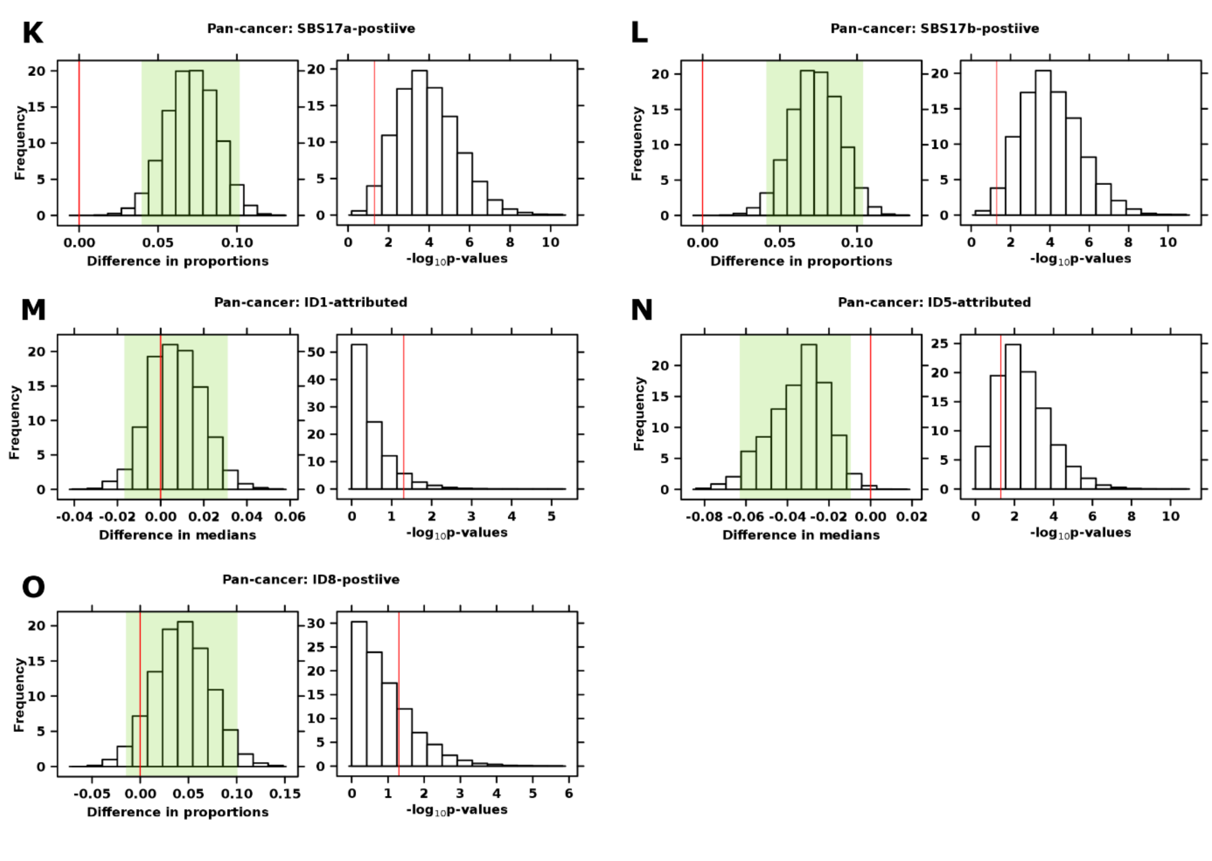

Supplementary Figure 2. Down-sampling on tumour subtype for pan-cancer analyses. Results from repeated down-sampling analysis of all pan-cancer findings to evaluate effect of overrepresented tumour subtypes. For each set of plots, the left plot shows distribution of differences between male and female proportions with green region indicating 95% confidence interval and red line indicating equal effects between the sexes. The right plot shows p-value histogram from proportions tests. Red line shows a p=0.05 threshold. Down-sampling was repeated 10,000 times. **(A)-(G)** and **(I)-(O)** show one set of down-sampling analyses each. **(H)** All down-sampling analyses for all 4,285 sex-biased CNAs in pan-cancer tumours. Green region shows union of confidence intervals for all genes tested.

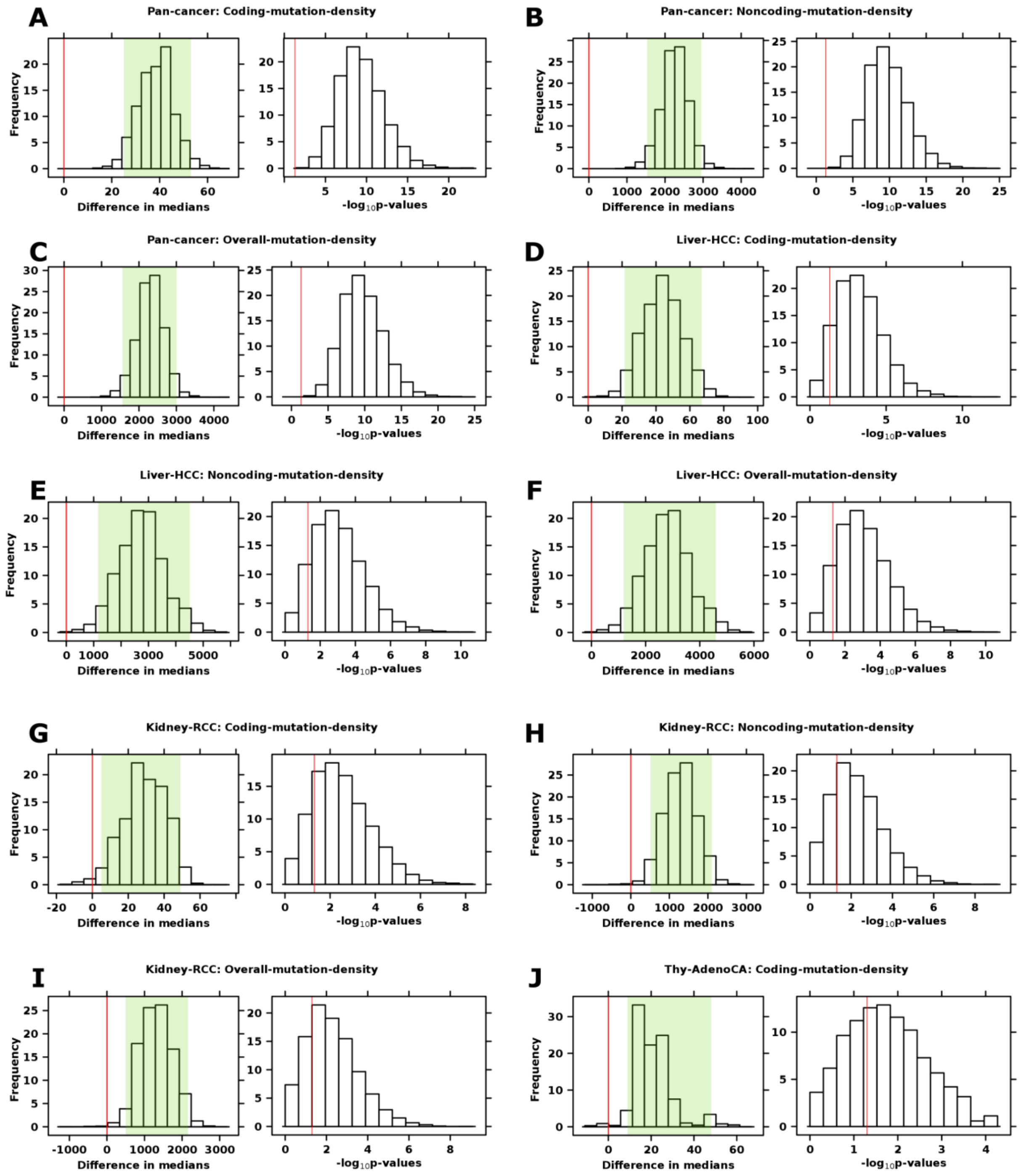

Supplementary Figure 3. Repeated down-sampling on sex for SNV density analysis. Results from repeated down-sampling analysis of SNV density findings to evaluate effect of imbalanced sex for **(A)-(C)** pan-cancer, **(D)-(F)** hepatocellular, **(G)-(I)** clear cell renal cell and **(J)** thyroid cancer. For each set of plots, the left shows distribution of differences between male and female proportions with green region indicating 95% confidence interval and red line indicating equal effects between the sexes. Right shows distribution of p-values from proportions tests with red line showing a p=0.05 threshold. Down-sampling was repeated 10,000 times.

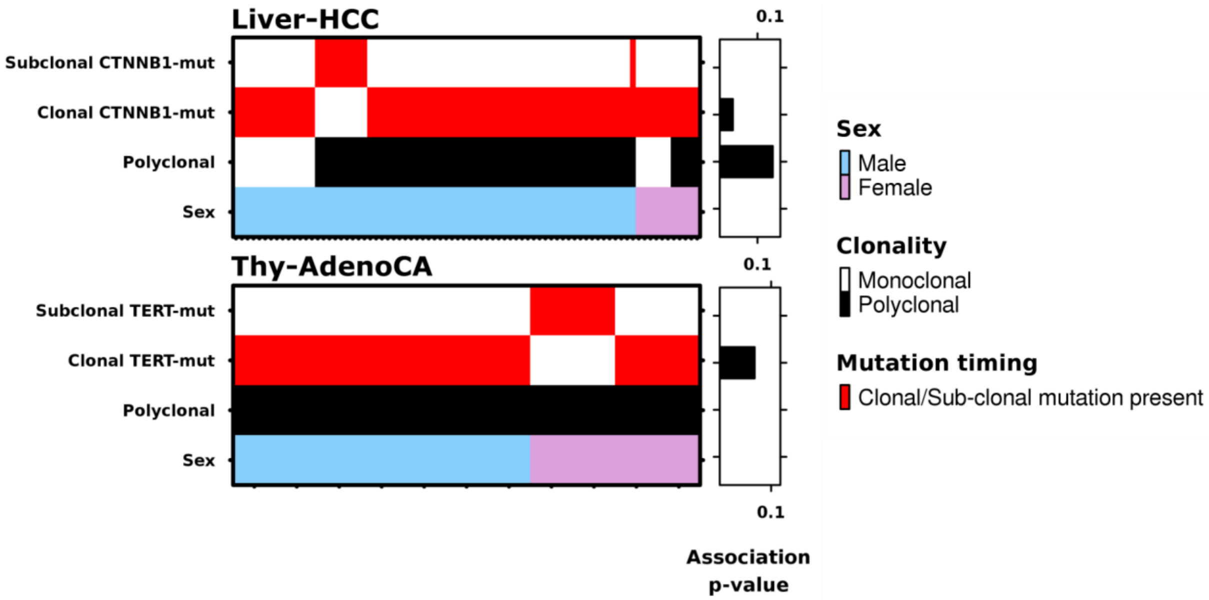

Supplementary Figure 4. Mutation timing of sex-biased driver events. For each driver event and tumour context, we show the association between sex and polyclonality status, presence of clonal mutations, and presence of subclonal mutations. Right plots show p-values for the association between sex and the row of interest as evaluated by proportion tests.

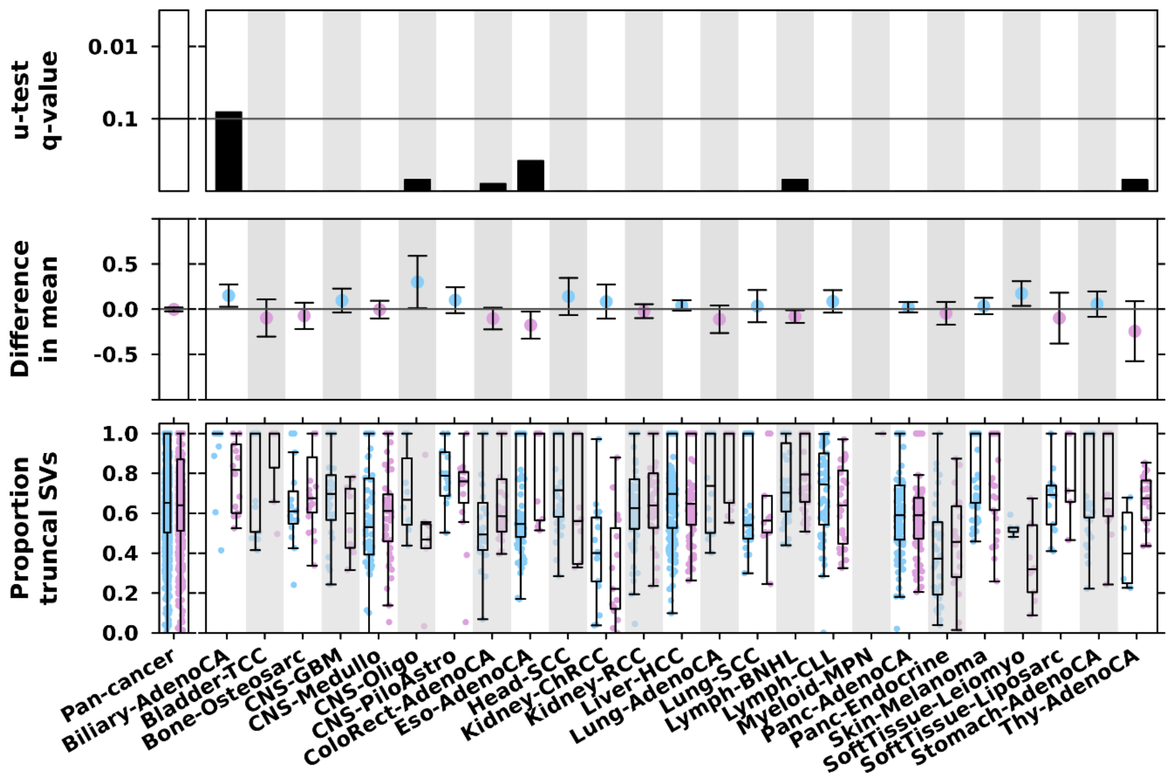

Supplementary Figure 5. Structural variation mutation timing is not found to be sex-biased. The proportion of truncal (*vs.* clonal) SVs is not statistically different between the sexes. From top to bottom: FDR-adjusted q-values from univariate Mann-Whitney *U*-tests with line denoting q = 0.1 threshold; difference in means for proportion of truncal SNVs with line denoting no differences in means; and boxplots for proportion of truncal SNVs for each sample, where blue shows male- and pink shows female-derived samples.

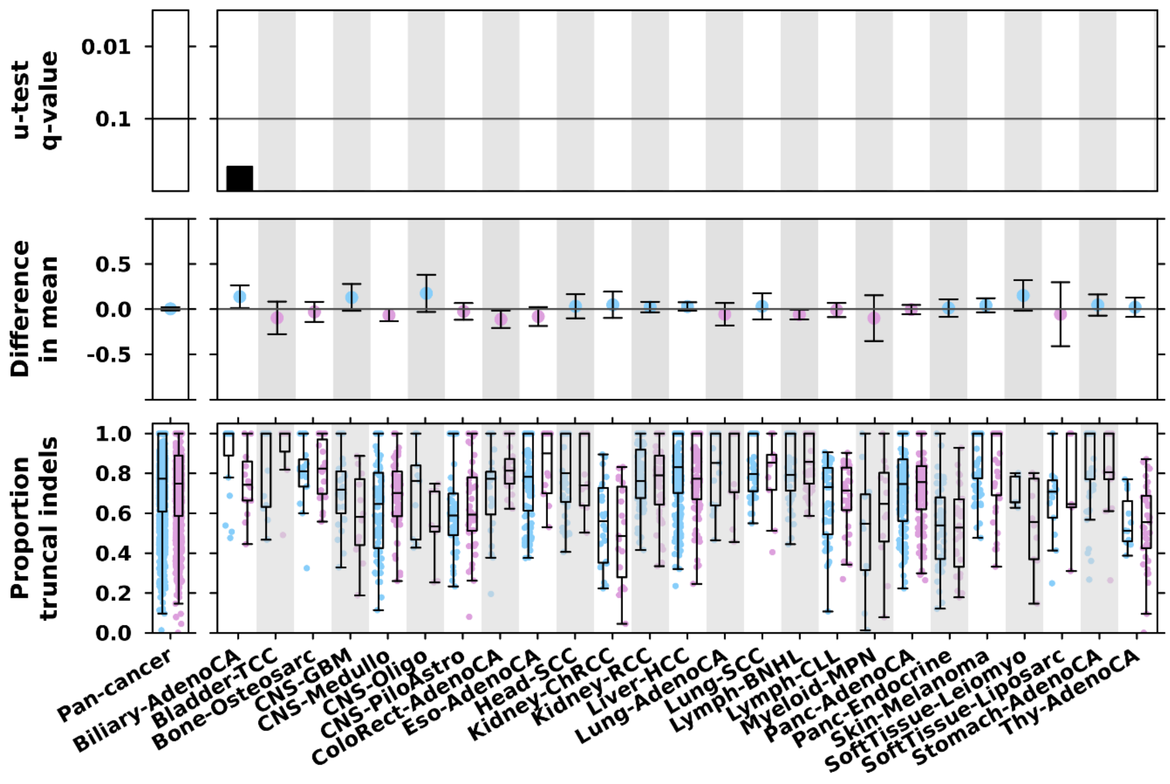

Supplementary Figure 6. Indel mutation timing is putatively sex-biased in CNS-Medullo. The proportion of truncal (*vs.* clonal) indels is univariately sex-biased in medulloblastoma. From top to bottom: FDR-adjusted q-values from univariate Mann-Whitney *U*-tests with line denoting q = 0.1 threshold; difference in means for proportion of truncal SNVs with line denoting no differences in means; and boxplots for proportion of truncal SNVs for each sample, where blue shows male- and pink shows female-derived samples.

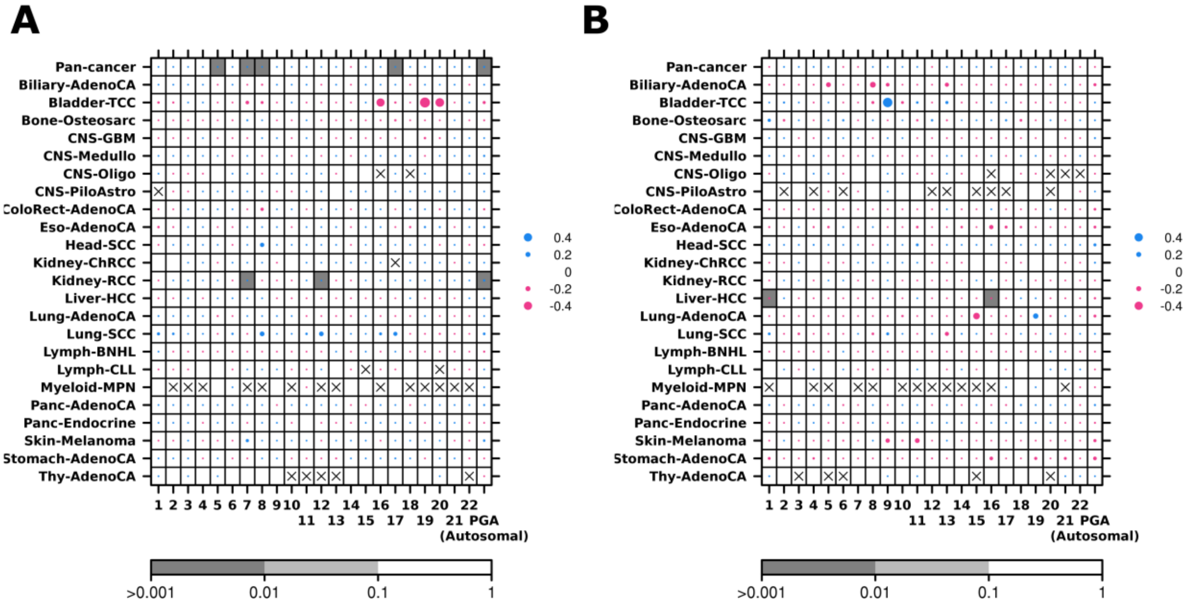

Supplementary Figure 7. Sex-biased percent copy altered in each tumour subtype. Summary of sex-biased **(A)** percent genome or chromosome gained and **(B)** percent genome or chromosome lost. Dot size shows difference in median percent genome gained/lost or percent chromosome gained/lost between the sexes. Dot colour shows direction of bias, with blue indicating higher instability in male-derived tumours and pink indicating higher instability in female-derived tumours. Background shading shows q-values from multivariate linear regression.

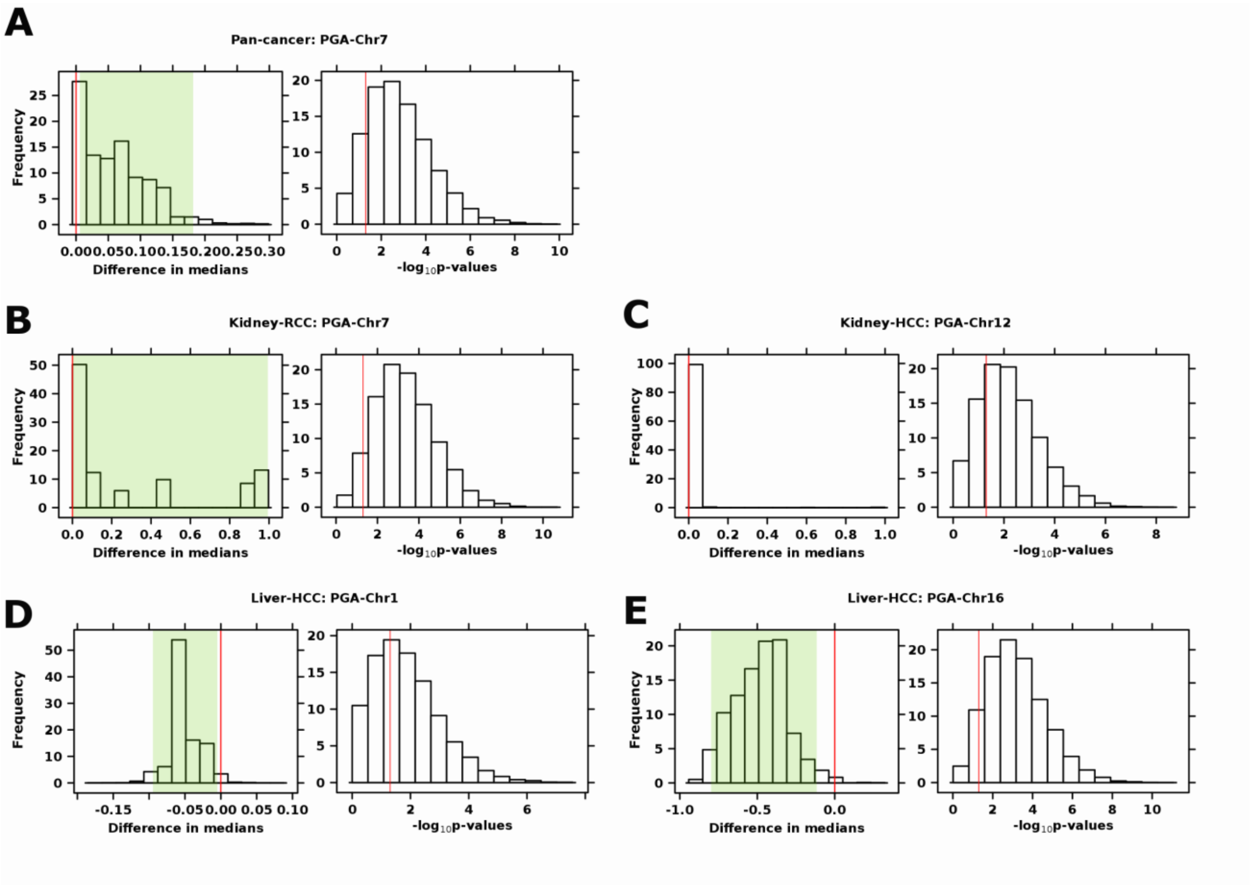

Supplementary Figure 8. Repeated down-sampling analysis on sex for PGA.

Results from repeated down-sampling analysis of genome instability findings to evaluate effect of imbalanced sex for **(A)** pan-cancer chromosome 7, clear cell renal cell cancer **(B)** chromosome 6 and **(C)** chromosome 12, and hepatocellular cancer **(D)** chromosome 1 and **(E)** chromosome 16. For each set of plots, the left plot shows distribution of differences between male and female medians with green region indicating 95% confidence interval and red line indicating equal effects between the sexes. Right plot shows p-value distribution from Mann-Whitney *U*-tests with red line showing a p=0.05 threshold. Down-sampling was repeated 10,000 times.

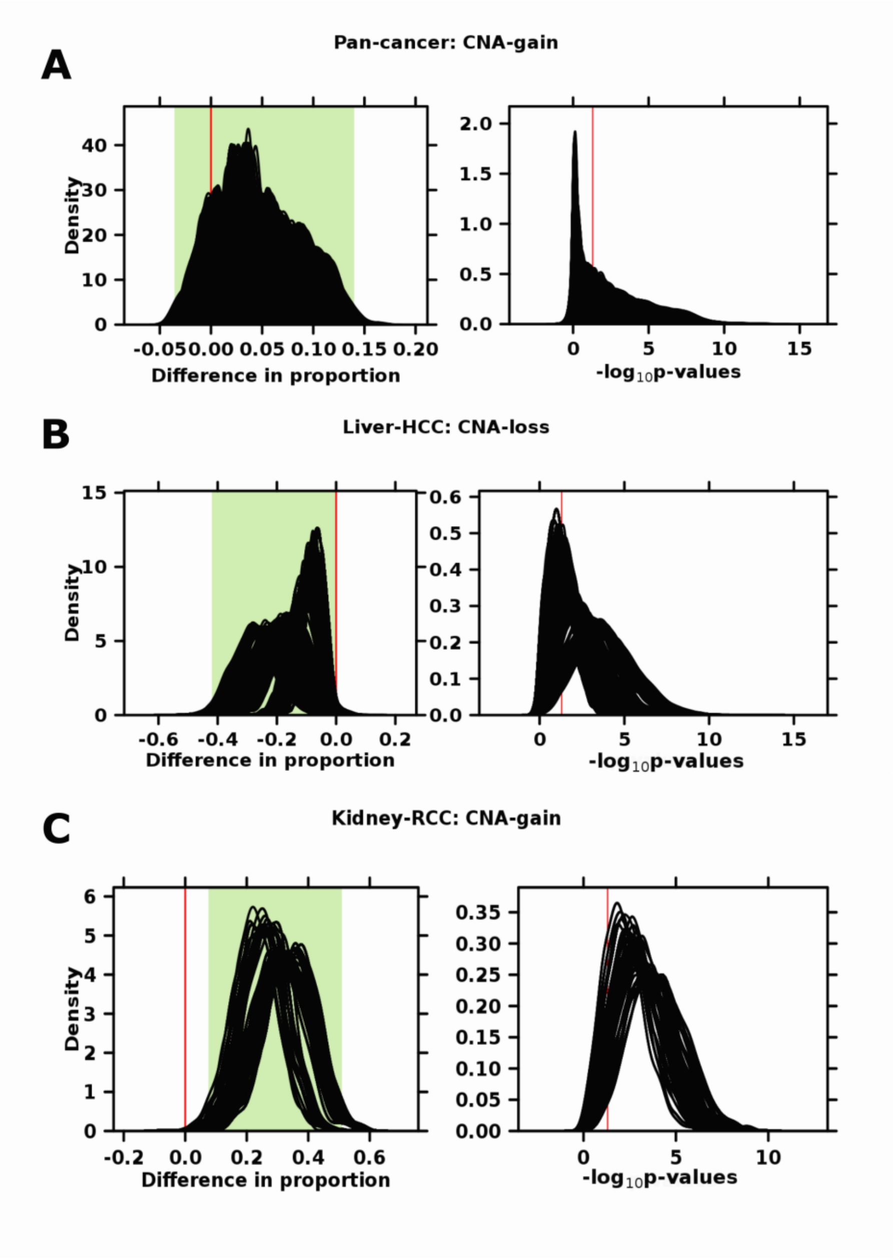

Supplementary Figure 9. Repeated down-sampling on sex for CNA findings.
Results from repeated down-sampling analysis of CNA findings to evaluate effect of imbalanced sex. Down-sampling was repeated 1,000 times per CNA event, and combined results are shown for all down-sampling repetitions for every CNA event. For each set of plots, left shows distribution of differences between male and female proportions for CNAs with red line indicating equal proportions between sexes and green region showing union of 95% confidence intervals for all genes tested. Right plot shows density of p-values from proportions test with red line indicating p=0.05 threshold. Each line in each plot shows the distribution of effect sizes and p-values for one CNA event. **(A)** Pan-cancer down-sampling results for 2,907 sex-biased gains. **(B)** Hepatocellular cancer down-sampling results for 2,078 sex-biased losses. **(C)** Renal cell cancer down-sampling results for 2,912 sex-biased gains.

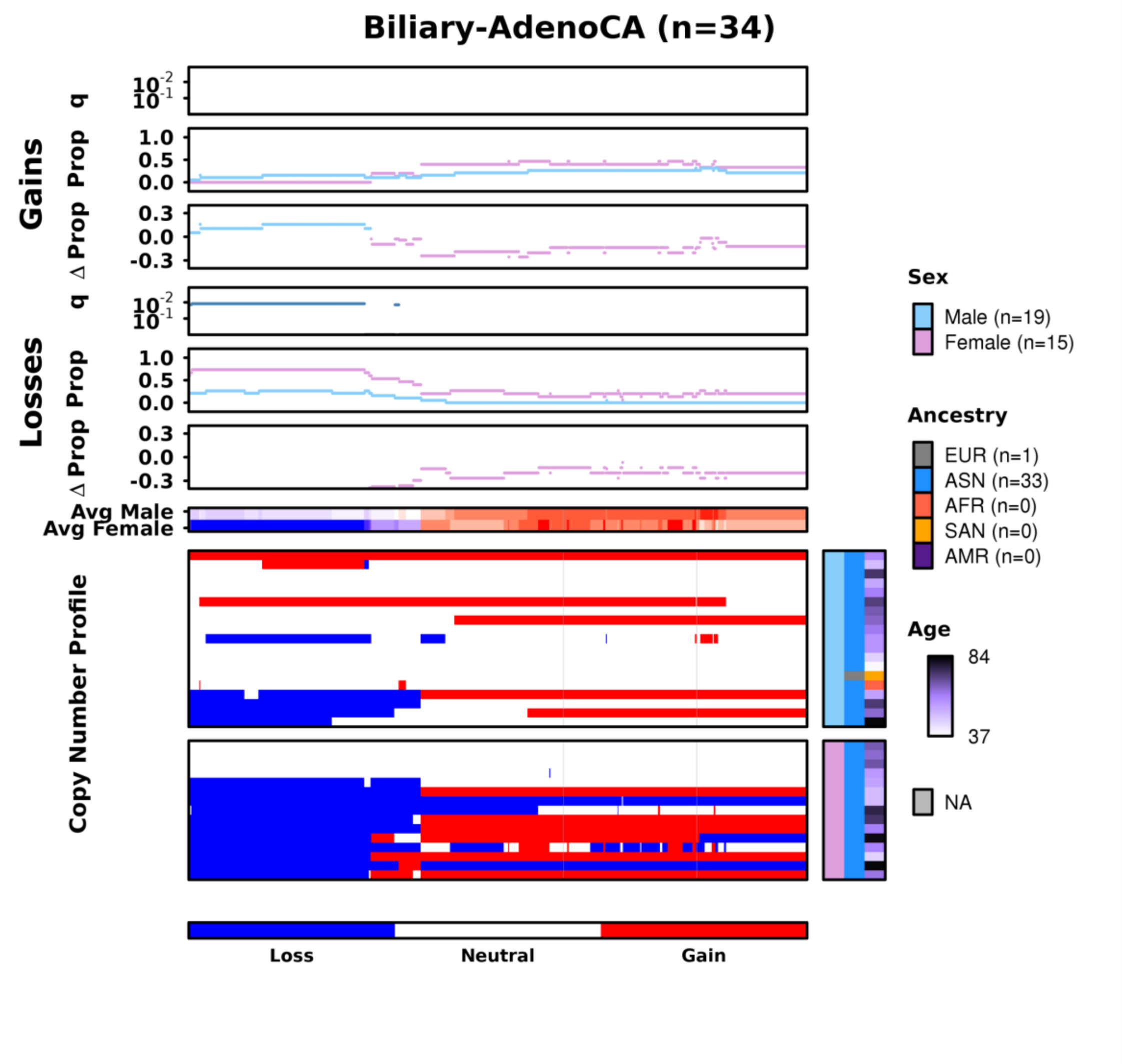

Supplementary Figure 10. Sex-biased CNAs in Biliary Adenocarcinoma from PGA-guided analysis. Each panel shows, from top to bottom: the q-value showing significance of sex from multivariate modeling on gene bins, proportion of samples with aberration, difference in proportion between male and female groups for amplifications; the same repeated for deletions and the CNA profile heatmap. The columns represent genes ordered by chromosome. The rows of the heatmap represent samples.

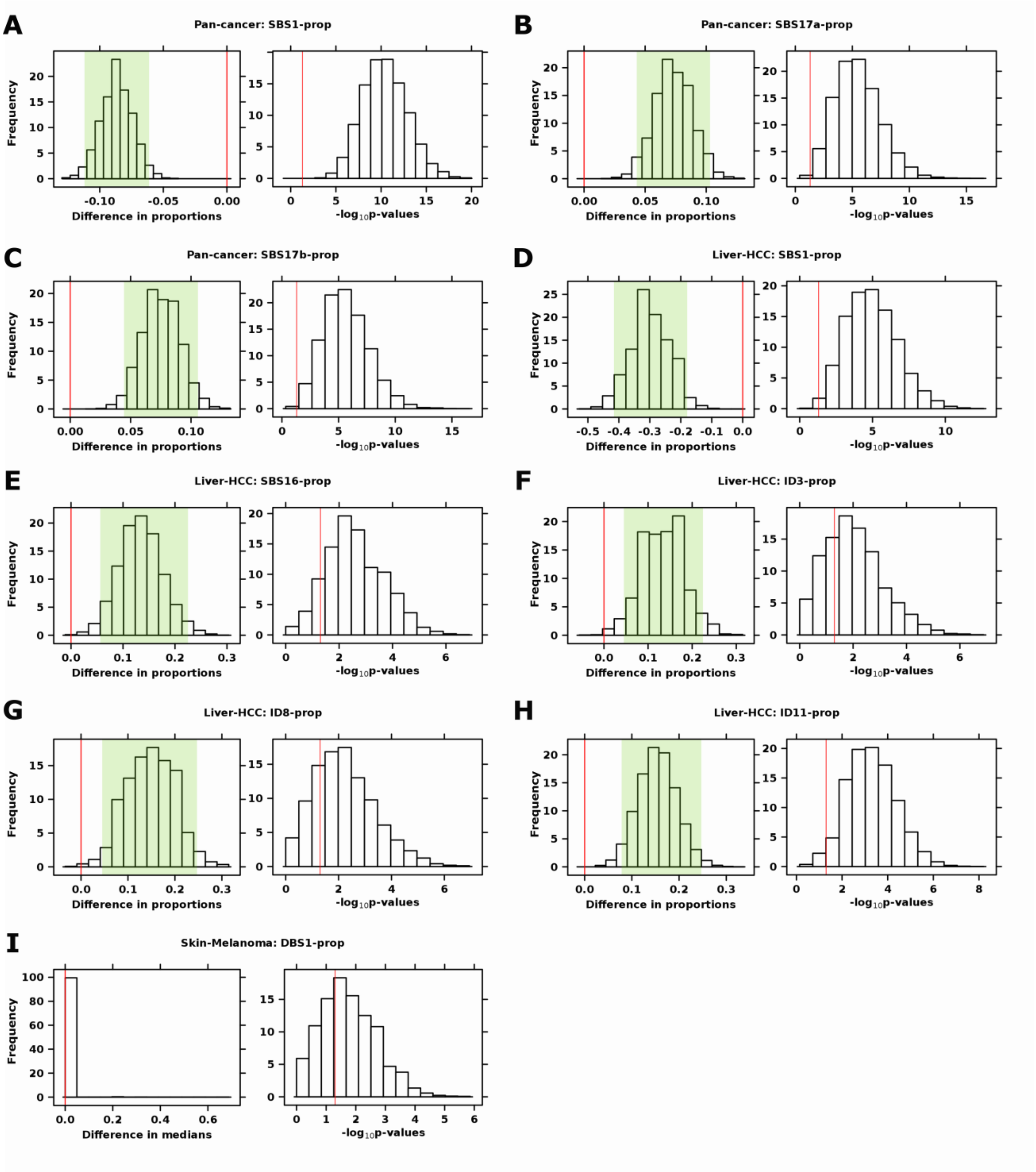

Supplementary Figure 11. Repeated down-sampling on sex for signature-positive analysis. Results from repeated down-sampling analysis of signature-positive findings to evaluate effect of imbalanced sex in **(A)-(C)** pan-cancer, **(D)-(H)** hepatocellular and **(I)** melanoma tumours. For each set of plots, the left plot shows distribution of differences between male and female proportions with green region indicating 95% confidence interval and red line indicating equal effects between the sexes. Right plot shows p-value distribution from proportion-tests with red line showing a p=0.05 threshold. Down-sampling was repeated 10,000 times for each sex-biased signature.

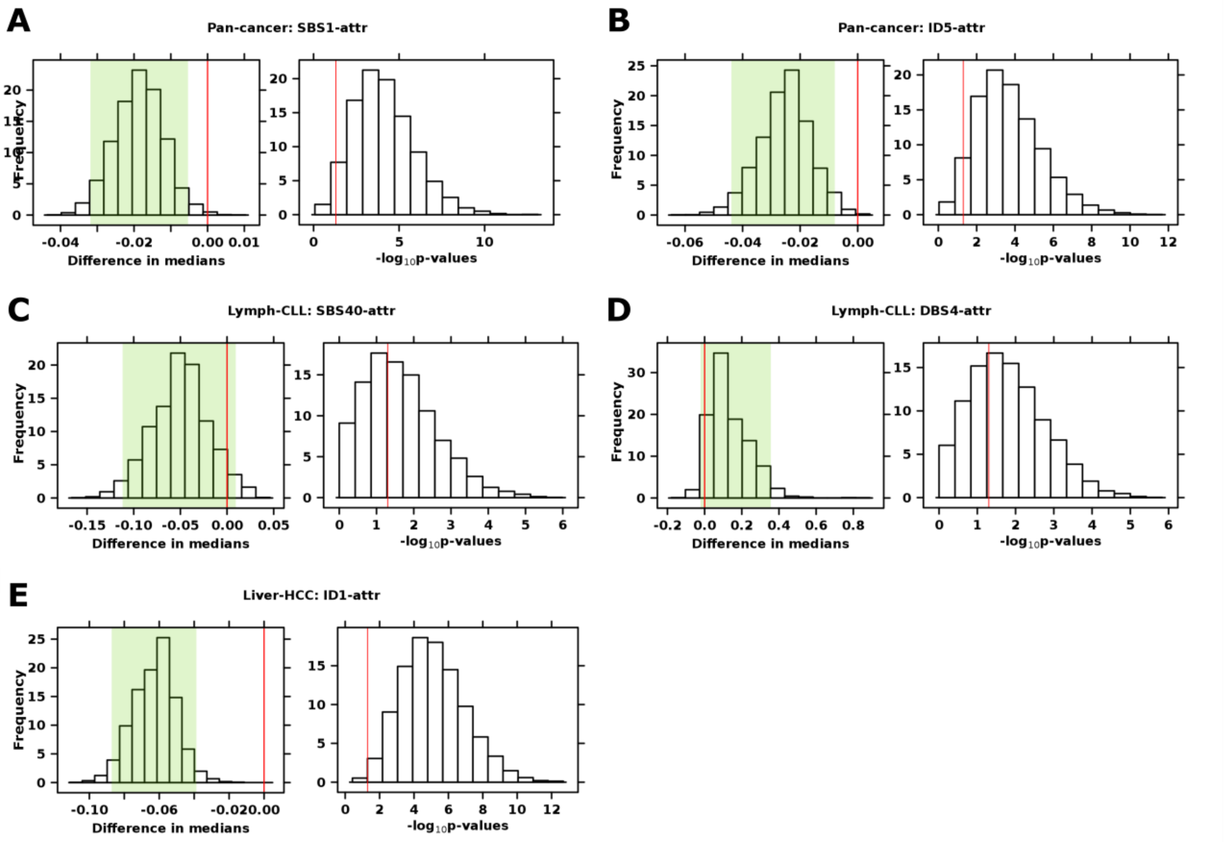

Supplementary Figure 12. Repeated down-sampling on sex for signature-attributed mutations analysis. Results from repeated down-sampling analysis of signature-attributed findings to evaluate effect of imbalanced sex in **(A)-(B)** pan-cancer, **(C)** chronic lymphocytic leukemia and **(D)-(E)** hepatocellular tumours. For each set of plots, the left plot shows distribution of differences between male and female median percentage of signature-attributed mutations. The green region indicates 95% confidence interval and red line indicates equal effects between the sexes. Right plot shows p-value distribution from Mann-Whitney *U*-tests with red line showing p=0.05 threshold. Down-sampling was repeated 10,000 times for each sex-biased signature.

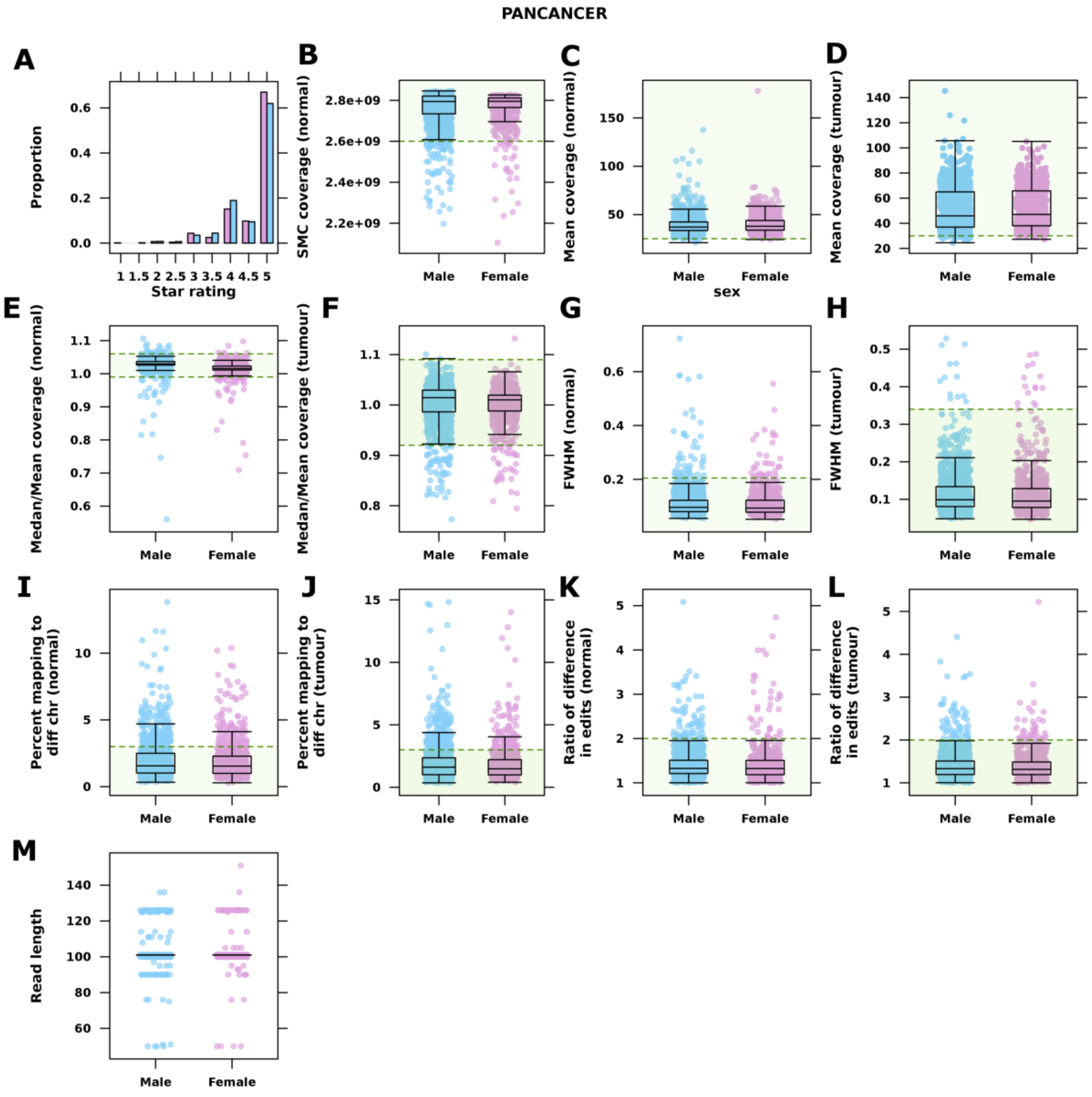

Supplementary Figure 13. Quality control metrics by sex for pan-cancer samples. Each plot shows the comparison between female- (pink) and male-derived (blue) samples for 13 quality control metrics assessing **(A)** general quality out of 5 stars, **(B)** somatic mutation calling coverage, **(C)-(F)** tumour and normal coverage, **(G)-(H)** full width at half maximum, **(I-J)** paired read mapping, **(K)-(L)** mapped read edits and **(M)** read length. Green regions indicate PCAWG quality-pass thresholds.
