# Supplementary material for: Sex differences in oncogenic mutational processes
Source: Nat Commun. 2020 Aug 28;11:4330. doi: 10.1038/s41467-020-17359-2 (PMC7455744; doi:10.1038/s41467-020-17359-2)
Supplement: Supplementary file 3 — Description of Additional Supplementary Files [file 41467_2020_17359_MOESM3_ESM.pdf]

Description of Additional Supplementary Files

File Name: Supplementary Data 1
Description: **Model & Data Summary**. Includes full description of variables included in multivariate models for all analyses, as well as breakdowns of variables for each tumour subtype.

File Name: Supplementary Data 2
Description: **Sex-Associated Driver Mutation Results.** Univariate and multivariate results for associations between sex and driver mutations for each tumour subtype.

File Name: Supplementary Data 3
Description: **Sex-Associated Single Nucleotide Variant Load Results.** Univariate and multivariate results for associations between sex and single nucleotide variant load for coding, non-coding and overall contexts.

File Name: Supplementary Data 4
Description: **Sex-Associated Measures of Tumour Evolution Results**.
Univariate and multivariate results for associations between sex and polyclonality, and between sex and mutation timing for single nucleotide variants, indels and structural variants.

File Name: Supplementary Data 5
Description: **Sex-Associated Genome Instability Results.** Univariate and multivariate results for associations between sex and percent genome altered. Comparisons provided for copy number alterations, gains and losses of each chromosome.

File Name: Supplementary Data 6
Description: **Sex-Associated Copy Number Loss Results.** Univariate and multivariate results for associations between sex and copy number loss of each gene for pan-cancer and tumour subtype results.

File Name: Supplementary Data 7
Description: **Sex-Associated Copy Number Gain Results.** Univariate and multivariate results for associations between sex and copy number gain of each gene for pan-cancer and tumour subtype results.

File Name: Supplementary Data 8
Description: **Sex-Associated Mutational Signatures Results**. Univariate and multivariate results for associations between sex and oncogenic mutational signatures. Results given for both proportion of signature positive tumours and signature activity.

File Name: Supplementary Data 9
Description: **Associations between Sex and Quality Control Metrics.** Summary of univariate and multivariate results for associations between sex and quality control metrics for sequencing and alignment.

File Name: Supplementary Data 10
Description: **Data Accession and Links.** Description of PCAWG data used in study with accession information through both Synapse and the ICGC data portal.
